# Exploring needs-based crafting in daily life: a registered report of a mixed-methods daily diary study

**DOI:** 10.1080/21642850.2025.2537699

**Published:** 2025-08-01

**Authors:** Martin Tušl

**Affiliations:** Public and Organizational Health, Center of Salutogenesis, Epidemiology, Biostatistics and Prevention, University of Zurich, Zurich, Switzerland

**Keywords:** needs-based crafting, psychological needs, well-being, diary study, mixed-methods

## Abstract

Employee well-being is essential for both individual fulfillment and organizational productivity. This registered report outlines a mixed-methods daily diary study designed to understand how employees use needs-based crafting strategies to fulfill their psychological needs across work and nonwork domains. Over a four-week period, 100 employees from diverse occupational sectors in Germany will participate. Data collection includes a baseline survey, daily diary assessments over two weeks, and a follow-up survey in the final week. The study captures both quantitative and qualitative data on daily crafting strategies, contextual influences, and individual differences. Quantitative analyses will examine within-person fluctuations and between-person differences in needs-based crafting and well-being indicators using multilevel modeling. Qualitative responses will be analyzed thematically and further explored for variations across employee subgroups defined by individual characteristics and well-being profiles. The study aims to uncover how employees actively shape their daily experiences to meet core psychological needs, how these strategies vary across individuals and contexts, and how they relate to employee well-being. Findings are expected to inform future interventions and practical recommendations for supporting employee well-being through needs-based crafting approaches in both work and nonwork life domains.

## Background

Modern work designs are characterized by increasing digitalization, emphasis on employee agility and flexibility, and a strong focus on job performance. These trends have been further accelerated by the profound disruptions of the COVID-19 crisis and the rapid integration of artificial intelligence into work processes (Gagné et al., 2022). Although flexible work designs offer employees greater opportunities for personal growth and autonomy, they also impose higher demands on self-management, personal initiative, and the proactive management of the interface between work and nonwork life (Shifrin & Michel, 2022). This becomes even more challenging due to blurring boundaries between life domains. Actions and experiences in employees’ work domain can significantly affect those in their nonwork domain, and vice versa (Sonnentag et al., 2017). Consequently, there is a need to equip employees with effective strategies to shape their work and nonwork activities and experiences in ways that support their well-being and performance in both life domains. This registered report outlines a study with a mixed-methods daily diary design to investigate needs-based crafting as a promising individual-level strategy for promoting employee well-being in both work and nonwork contexts (de Bloom et al., 2020).

### Employees as active agents of their work and nonwork life: moving beyond job crafting research

Traditionally, job design theories had a top-down perspective, in which employees are seen as passive recipients of work characteristics created by the employers and management (e.g. Demerouti et al., 2001; Hackman & Oldham, 1976). However, this perspective falls short in explaining why there is such a rich variety of working conditions of individuals who hold the same jobs, even in the same organization (Bakker & Demerouti, 2017). Over two decades ago, the emergence of *job crafting* introduced a new bottom-up perspective to job design theories. Job crafting refers to employees’ proactive, intentional, and goal-oriented efforts to align their job with their needs, skills, and preferences (Wrzesniewski & Dutton, 2001). Since the introduction of the term, job crafting has become a prominent topic in occupational research generating multiple theoretical conceptualizations and quantitative scales to measure job crafting and crafting in other life domains (Tims et al., 2022). The accumulated evidence thus far has shown that job crafting is related to a wide variety of positive outcomes for employees such as work engagement, job satisfaction, and job performance (Rudolph et al., 2017; Zhang & Parker, 2019). Currently, most quantitative measures in job crafting research are based on the job demands-resources model, focusing on employee-initiated changes to external job demands and resources (Bakker & Demerouti, 2017; Bindl et al., 2019; Tims et al., 2012). While these measures are well-suited for assessing pre-defined job crafting behaviors, they do not capture the wide variety of possible employee crafting strategies used across different occupational groups. In addition, the model’s theoretical focus on the workplace context (Bakker & Demerouti, 2017) limits the applicability of the scales to nonwork contexts beyond the demands-resources perspective (e.g. Demerouti et al., 2019). A new line of integrative crafting research, referred to as needs-based crafting, addresses these challenges by emphasizing psychological needs as the primary drivers of employee crafting efforts that are relevant within different contexts across work and nonwork domains (de Bloom et al., 2020).

### Needs-based crafting: psychological needs at the center of employee crafting efforts

Psychological needs are intrinsic motivational forces that drive human behavior, connecting various aspects of life and aligning individual motives and efforts (Ryan & Deci, 2000). Self-Determination Theory (Ryan & Deci, 2000) is the most prominent framework explicitly theorizing autonomy, competence, and relatedness as basic psychological needs. However, other influential frameworks, such as the Effort-Recovery Model (Meijman & Mulder, 1998), recovery experiences (Sonnentag & Fritz, 2007), the concept of flow (Csikszentmihalyi, 1990), or Frankl’s will to meaning (1984), emphasize core motivational mechanisms relevant to psychological needs. Although they differ in focus, they all share the fundamental idea that fulfilling certain psychological needs supports human well-being (Ryan & Deci, 2000).

Building on this foundation, Newman and colleagues proposed the DRAMMA model – Detachment, Relaxation, Autonomy, Mastery, Meaning, and Affiliation – as a comprehensive framework for understanding how leisure experiences enhance well-being (Newman et al., 2014). Detachment and relaxation restore energy by reducing physical and/or mental strain, while autonomy, mastery, meaning, and affiliation promote positive states, personal growth, and resources development. Empirical studies have supported the relevance of the DRAMMA needs for employee well-being (Kujanpää et al., 2021; Virtanen et al., 2020).

The needs-based model of crafting builds on this perspective by proposing that employees proactively shape their experiences to satisfy psychological needs across both work and nonwork domains. This shift from job demands and resources to intrinsic needs offers several advantages (de Bloom et al., 2020). This explicit shift from demands and resources to psychological needs implies several important strengths. First, it broadens the range of relevant strategies across life domains. For example, one might fulfill the need for affiliation by organizing a family gathering outside work or engaging in casual conversations with colleagues during the workday. Second, the needs-based model explains individual differences in the crafting strategies employees use to fulfill their needs (de Bloom et al., 2020). For example, one employee may take on a new project to gain more control as social bonds with colleagues (i.e. crafting for affiliation). Motivation for engaging in the same crafting activity may also shift over time within an individual, highlighting the dynamic, goal-oriented nature of crafting and the central role of intrinsic motivation.

Recently, quantitative scales have been developed to assess needs-based crafting in both work and nonwork contexts integrating the needs-based model of crafting (de Bloom et al., 2020) and the DRAMMA needs as their foundation (Tušl et al., 2024). Initial findings from empirical studies indicate the positive impact of needs-based crafting on outcomes such as work engagement, recovery, life satisfaction, family role performance, perceived work ability, and daily energy levels (Kosenkranius et al., 2023; Kujanpää et al., 2022; Tušl et al., 2022; Tušl et al., 2024).

### Present study

The needs-based crafting scales (Kujanpää et al., 2022; Tušl et al., 2024) enable researchers to assess *if* and *how often* employees proactively shape their activities to satisfy their DRAMMA needs (e.g. off-job crafting for mastery: ‘I’ve arranged my off-job activities so that I experience proficiency in the things I undertake’). A major strength of these scales is that they let respondents define their own strategies. However, this flexibility also creates a ‘black box’ regarding the specific ways people craft to meet their needs. Opening this black box is a great opportunity for identifying which strategies are most effective in specific contexts and for designing targeted interventions. Therefore, the present study will investigate the diverse crafting strategies employees engage in across both work and nonwork domains, as well as their day-to-day dynamics. Using a mixed-methods daily diary design, the study will generate both qualitative and quantitative data to explore how employees fulfill their psychological needs in everyday life.

### Study objectives

The study aims to examine both within-person and between-person processes of needs-based crafting. At the within-person level, it focuses on the day-to-day dynamics of needs-based crafting across work and nonwork settings, while the between-person level investigates how stable characteristics (e.g. sociodemographics, personality traits, work charactereistics) influence these crafting efforts. To achieve this, the study has the following six specific objectives:

1. Investigate the day-to-day variability and associations between needs-based crafting and well-being within individuals.
2. Identify and categorize specific crafting strategies employees use to fulfill their DRAMMA psychological needs in both work and nonwork contexts.
3. Examine how stable individual and contextual characteristics (e.g. sociodemographic factors, work characteristics) influence employee crafting strategies.
4. Examine how situational factors (e.g. work vs. nonwork settings, day of the week) influence crafting strategies and how these factors vary across DRAMMA needs.
5. Analyze crafting strategies among employees with different latent profiles based on well-being indicators (e.g. Do highly engaged employees use different or more varied crafting strategies?).
6. Develop practical recommendations, including illustrative examples of effective needs-based crafting strategies tailored to specific occupational groups (e.g. healthcare, education, manual labor).

## Methods

### Participants

The study targets a sample of approximately 100 employees from Germany across a variety of occupational groups. The target sample is sufficiently large to allow for within- and between-person quantitative analyses of daily diary data, including multilevel modeling and latent profile comparisons, while remaining small enough to enable an in-depth qualitative examination of individual crafting strategies that is central to this study. Participants will be recruited through a professional panel data provider using the following eligibility criteria: full-time employment, age between 18–65 years, fluency in German, employment in Germany, and not self-employed. The target sample aims to represent a diverse range of employees in Germany based on variations in sociodemographic and occupational characteristics.

### Measures and data collection

The main part of the study uses a mixed-methods design with daily diary assessments. Data will be collected electronically via the SoSci Survey platform, accessible with a smartphone or a computer, and hosted on a secure university server. Upon recruitment, participants will receive detailed information outlining the study’s objectives, procedures, potential risks and benefits, and their rights as research participants. They will provide informed consent prior to participation. Each participant will be assigned a unique code by the panel provider to ensure anonymized identification throughout all stages of data collection. Survey links and reminders will be sent to participants via email by the panel provider. Data collection is scheduled to begin in April 2025^1^ and will consist of three parts over a four-week period:

1. *Baseline questionnaire.* In the first week, participants will report individual and contextual characteristics including gender, age, education level, personality traits (Rammstedt & John, 2007), living situation, caregiving responsibilities, and job-related characteristics (e.g. type of occupation, job demands and resources). The baseline survey will include measures of needs-based crafting (Kujanpää et al., 2022; Tušl et al., 2024) and standardized well-being indicators including the mental health continuum (Keyes, 2002), work engagement (Schaufeli et al., 2006), recovery experiences (Sonnentag & Fritz, 2007), and burnout complaints (Schaufeli et al., 2020).
2. *Diary study*. The main part of the study will run in the second and third week with the first measurement on Monday and the last measurement on Sunday. Each day at 18h, participants will receive a brief survey (2–4 minutes), which they can complete anytime between 18h and 23h. This will yield 14 responses per person resulting in approximately 1400 data points for the full sample. The design will allow to capture a diverse range of crafting strategies and behaviors across work and nonwork life contexts and to account for day-of-week variability. The daily survey will include:
  1. An item that determines whether the participant worked for at least four hours that day to distinguish between work and nonwork contexts (i.e. ‘Did you work today (at least four hours)?’).
  2. Daily well-being measures: mental well-being (e.g. ‘Today, I’ve been feeling optimistic about the future’; Tennant et al., 2007), daily energy levels (Weigelt et al., 2022); and for working days brief measures of work engagement with three items (e.g. ‘Today, I felt bursting with energy at my work’; Schaufeli et al., 2006) and burnout complaints with four items (e.g. ‘Today, I struggled to find any enthusiasm for my work’; Schaufeli et al., 2020). The measures will use answering format from 1 =  *Strongly disagree* to 6 = *Strongly agree*.
  3. A quantitative measure of needs-based crafting (Kujanpää et al., 2022; Tušl et al., 2024) will be used to assess daily crafting efforts aimed at satisfying DRAMMA needs in either the work or nonwork context. The short version consists of six items selected from the validated 18-item needs-based crafting scale, based on high factor loadings on each DRAMMA dimension. Each item targets one specific need (e.g. job crafting for affiliation: ‘Today, I’ve made sure to experience close connections to the people around me at work’). The six items are parallel in structure across the work and nonwork versions of the scale. The answering format is from 1 =  *Strongly disagree* to 6 = *Strongly agree*. For responses indicating agreement (ratings of 4–6), an open-ended follow-up will prompt participants to describe the specific strategies they used to satisfy the particular need(s):
  4. ‘You indicated that today you actively shaped your work/nonwork activities to fulfill the following psychological needs: ‘*a list of DRAMMA needs based on participant’s answer*’. Please briefly describe the specific activities or strategies you used to actively fulfill these psychological needs (10–100 words). *Note: You can use the voice-to-text function on your smartphone to input your response by voice instead of typing it. This is usually located next to the space bar on your keyboard.*’
  5. The open-ended item will be supported by an optional voice-to-text transcription feature, allowing participants to provide responses via a voice message, which will be automatically transcribed. This flexible approach is expected to increase adherence and enrich the qualitative data (Haag et al., 2023).
3. *Final questionnaire.* Participants will receive a follow-up questionnaire three days after completing the diary phase, containing the same needs-based crafting and well-being measures as in the baseline. As the diary study prompts participants to reflect on their work and well-being, comparing baseline and follow-up data will allow for the assessment of potential changes over time.

### Data analysis

To address the objectives of the study, the following approaches for between- and within-person analyses will be applied*:*

*Between-person*: Qualitative data from open-ended diary responses will be analyzed using thematic analysis (Braun & Clarke, 2006), applying an abductive analytic strategy. The analysis will be guided by the DRAMMA needs (Newman et al., 2014) as a structure for the coding framework. Within these predefined categories, an inductive process will be used to identify the specific crafting strategies participants describe. This abductive approach enables us to integrate theory-driven categories with emergent, data-driven insights into how employees actively craft for psychological need satisfaction. In the next step, these qualitative categories will be examined in relation to individual and contextual characteristics collected at baseline (e.g. sociodemographic and work characteristics) to explore variations in needs-based crafting strategies across different employee subgroups. Finally, qualitative data will be linked with baseline and follow-up quantitative indicators of well-being. Latent profile analysis will be conducted using continuous measures of well-being to identify distinct participant profiles based on shared response patterns in well-being and crafting indicators (Morin et al., 2018). These profiles will be then examined for variations in crafting strategies to provide deeper insights into the interplay between specific crafting strategies and employee well-being (Mäkikangas, 2018).

*Within-person*: To examine within-person dynamics, the daily quantitative data will be analyzed using multilevel mixed-effects modeling and 1-day lagged models, accounting for the nested structure of days within individuals. This approach will allow for the assessment of both, concurrent associations (i.e. same-day needs-based crafting and same-day well-being outcomes) and temporal associations (i.e. whether needs-based crafting on one day predicts well-being on the following day). In a final integrative step, insights from the qualitative data will be linked with these daily quantitative patterns to explore how specific strategies correspond with short-term well-being outcomes. Quantitative analyses will be conducted in R (R Core Team, 2024), using the *tidyverse* package (Wickham et al., 2019) for data handling, and *lme4* (Bates et al., 2015) and *emmeans* (Lenth, 2024) packages for linear mixed-effects modeling. In addition, Mplus (Muthén & Muthén, 2017) will be used to estimate lagged models.

## Expected results

The study will provide unique evidence about the crafting strategies employees use day-to-day across different work and nonwork contexts and their relation to well-being outcomes. Apart from the theoretical and empirical insights, the study will develop practical recommendations with specific examples of employee crafting strategies for different occupational groups. Findings of the study aim to support individual crafting efforts, organizational well-being initiatives, and the design of targeted crafting interventions.

## Discussion

The study will contribute to the field of occupational health psychology by addressing key gaps in our understanding of employee crafting efforts. Specifically, it aims to open ‘a black box’ by identifying the specific strategies employees use to fulfill psychological needs across both work and nonwork domains. Additionally, by examining variations in crafting strategies across employee subgroups, the study will shed light on how individual characteristics and contextual factors shape crafting efforts – an area that has received only limited empirical attention to date (Rudolph et al., 2017). Finally, by profiling employees based on quantitative indicators of well-being, the study will generate evidence on effective crafting strategies for fostering well-being. The study will provide detailed insights into the ways in which needs-based crafting impacts employee well-being on a day-to-day basis in different occupational contexts.

From a practical perspective, the findings have the potential to benefit organizations, managers, and employees alike. The insights can help employees proactively shape their work and nonwork lives in ways that support sustainable well-being. The evidence will also inform the development of targeted interventions to promote effective crafting strategies within organizational settings. Overall, the study will provide a unique and innovative contribution to both the theory and practice of employee proactivity and well-being.

### Risk analysis

The study protocol was developed with several practical challenges in mind – participant compliance across the diary study, managing missing data, and minimizing participant burden. To address these, we limited the diary assessments to one measurement on each day instead of multiple measurements across only selected days. This will increase the variability of days covered by the study, while keeping the effort required relatively low. All survey items are mandatory, which will prevent accidental skipping and reduce missing data. Participants will receive a small incentive in the amount of 12€ for their participation in the study if they complete at least 80% of the daily surveys. Additionally, we will recruit 180 participants at baseline to account for potential dropouts and ensure that at least 100 complete the full study. These measures are intended to balance data quality with feasibility and to support a good effort-reward perception among participants.

## Data Availability

Data will be provided on the OSF platform, where the study is preregistered.

## Note

1 Data collection took place in May 2025, while the manuscript was still under peer review. The baseline questionnaire was administered between April 30th and May 4th. The daily diary phase ran from May 5th to May 18th, followed by the final survey, which was conducted from May 21st to May 25th.

## Open Scholarship

This article has earned the Center for Open Science badges for Open Materials. The materials are openly accessible at https://osf.io/azn2b/?view_only=707c37ab6bff46db94df30bc8473f75c.

This article has earned the Center for Open Science badges for Preregistered. The materials are openly accessible at https://osf.io/azn2b/?view_only=707c37ab6bff46db94df30bc8473f75c.

This article has earned the Center for Open Science badges for Preregistered+. The materials are openly accessible at https://osf.io/azn2b/?view_only=707c37ab6bff46db94df30bc8473f75c.

## Preregistation

osf.io/azn2b/

## References

[CIT0001] Bakker, A. B., & Demerouti, E. (2017). Job demands-resources theory: Taking stock and looking forward. *Journal of Occupational Health Psychology*, *22*(3), 273–285. 10.1037/ocp0000056 27732008

[CIT0002] Bates, D., Mächler, M., Bolker, B., & Walker, S. (2015). Fitting linear mixed-effects models using lme4. *Journal of Statistical Software*, *67*(1), 1–48. 10.18637/jss.v067.i01

[CIT0003] Bindl, U. K., Unsworth, K. L., Gibson, C. B., & Stride, C. B. (2019). Job crafting revisited: Implications of an extended framework for active changes at work. *Journal of Applied Psychology*, *104*(5), 605–628. 10.1037/apl0000362 30407042

[CIT0004] Braun, V., & Clarke, V. (2006). Using thematic analysis in psychology. *Qualitative Research in Psychology*, *3*(2), 77–101. 10.1191/1478088706qp063oa

[CIT0005] Csikszentmihalyi, M. (1990). *Flow: The psychology of optimal experience*. Harper & Row.

[CIT0006] de Bloom, J., Vaziri, H., Tay, L., & Kujanpää, M. (2020). An identity-based integrative needs model of crafting: Crafting within and across life domains. *Journal of Applied Psychology*, *105*(12), 1423–1446. 10.1037/apl0000495 32202815

[CIT0007] Demerouti, E., Bakker, A. B., Nachreiner, F., & Schaufeli, W. B. (2001). The job demands-resources model of burnout. *Journal of Applied Psychology*, *86*(3), 499–512. 10.1037/0021-9010.86.3.499 11419809

[CIT0008] Demerouti, E., Hewett, R., Haun, V., De Gieter, S., Rodríguez-Sánchez, A., & Skakon, J. (2019). From job crafting to home crafting: A daily diary study among six European countries. *Human Relations*, *73*(7), 1010–1035. 10.1177/0018726719848809

[CIT0009] Frankl, V. E. (1984). *Man’s search for meaning: An introduction to logotherapy*. Simon & Schuster.

[CIT0010] Gagné, M., Parker, S. K., Griffin, M. A., Dunlop, P. D., Knight, C., Klonek, F. E., & Parent-Rocheleau, X. (2022). Understanding and shaping the future of work with self-determination theory. *Nature Reviews Psychology* *1*(7), 378–392. 10.1038/s44159-022-00054-4. PMC9088153 35574235

[CIT0011] Haag, C., Steinemann, N., Chiavi, D., Kamm, C. P., Sieber, C., Manjaly, Z.-M., Horváth, G., Ajdacic-Gross, V., Puhan, M. A., & von Wyl, V. (2023). Blending citizen science with natural language processing and machine learning: Understanding the experience of living with multiple sclerosis. *PLOS Digital Health*, *2*(8), e0000305. 10.1371/journal.pdig.0000305 37531365 PMC10395829

[CIT0012] Hackman, J. R., & Oldham, G. R. (1976). Motivation through the design of work: Test of a theory. *Organizational Behavior and Human Performance*, *16*(2), 250–279. 10.1016/0030-5073(76)90016-7

[CIT0013a] Keyes, C. L. M. (2002). The mental health continuum: From languishing to flourishing in life. *Journal of Health and Social Behavior*, *43*(2), 207–222. 10.2307/3090197 12096700

[CIT0013] Kosenkranius, M., Rink, F., Weigelt, O., & de Bloom, J. (2023). Crafting and human energy: Needs-based crafting efforts across life domains shape employees’ daily energy trajectories. *Journal of Occupational Health Psychology*, *28*(3), 192–204. 10.1037/ocp0000347 36996161

[CIT0014] Kujanpää, M., Syrek, C., Lehr, D., Kinnunen, U., Reins, J. A., & De Bloom, J. (2021). Need satisfaction and optimal functioning at leisure and work: A longitudinal validation study of the DRAMMA model. *Journal of Happiness Studies*, *22*(2), 681–707. 10.1007/s10902-020-00247-3

[CIT0015] Kujanpää, M., Syrek, C., Tay, L., Kinnunen, U., Mäkikangas, A., Shimazu, A., Wiese, C. W., Brauchli, R., Bauer, G. F., Kerksieck, P., Toyama, H., & de Bloom, J. (2022). Needs-based off-job crafting across different life domains and contexts: Testing a novel conceptual and measurement approach. *Frontiers in Psychology*, *13*. 10.3389/fpsyg.2022.959296 PMC9536339 36211856

[CIT0016] Lenth, R. V. (2024). *emmeans: Estimated marginal means, aka least-squares means* (R package version 1.10.0). https://CRAN.R-project.org/package=emmeans.

[CIT0017] Mäkikangas, A. (2018). Job crafting profiles and work engagement: A person-centered approach. *Journal of Vocational Behavior*, *106*, 101–111. 10.1016/j.jvb.2018.01.001

[CIT0018] Meijman, T. F., & Mulder, G. (1998). Psychological aspects of workload. In C. De Wolff, P. J. D. Drenth, & T. Henk (Eds.), *Handbook of work and organizational: Work psychology* (Vol. 2, 2nd ed, pp. 5–33). Psychology Press/Erlbaum (UK) Taylor & Francis.

[CIT0019] Morin, A. J., Bujacz, A., & Gagné, M. (2018). Person-centered methodologies in the organizational sciences: Introduction to the feature topic. *Organizational Research Methods*, *21*(4), 803–813. 10.1177/1094428118773856

[CIT0020] Muthén, L. K., & Muthén, B. O. (2017). *Mplus: Statistical analysis with latent variables: User's guide (Version 8)*. Authors.

[CIT0021] Newman, D. B., Tay, L., & Diener, E. (2014). Leisure and subjective well-being: A model of psychological mechanisms as mediating factors. *Journal of Happiness Studies*, *15*(3), 555–578. 10.1007/s10902-013-9435-x

[CIT0022] Rammstedt, B., & John, O. P. (2007). Measuring personality in one minute or less: A 10-item short version of the big five inventory in English and German. *Journal of Research in Personality*, *41*(1), 203–212. 10.1016/j.jrp.2006.02.001

[CIT0023] R Core Team. (2024). *R: A language and environment for statistical computing*. R Foundation for Statistical Computing. https://www.R-project.org/.

[CIT0024] Rudolph, C. W., Katz, I. M., Lavigne, K. N., & Zacher, H. (2017). Job crafting: A meta-analysis of relationships with individual differences, job characteristics, and work outcomes. *Journal of Vocational Behavior*, *102*, 112–138. 10.1016/j.jvb.2017.05.008

[CIT0025] Ryan, R. M., & Deci, E. L. (2000). Self-determination theory and the facilitation of intrinsic motivation, social development, and well-being. *American Psychologist*, *55*(1), 68–78. 10.1037/0003-066X.55.1.6. 11392867

[CIT0026] Schaufeli, W. B., Bakker, A. B., & Salanova, M. (2006). The measurement of work engagement with a short questionnaire: A cross-national study. *Educational and Psychological Measurement*, *66*(4), 701–716. 10.1177/0013164405282471

[CIT0027] Schaufeli, W. B., Desart, S., & De Witte, H. (2020). Burnout assessment tool (Bat) – development, validity, and reliability. *International Journal of Environmental Research and Public Health*, *17*(24), 1–21. 10.3390/ijerph17249495 PMC7766078 33352940

[CIT0028] Shifrin, N. V., & Michel, J. S. (2022). Flexible work arrangements and employee health: A meta-analytic review. *Work & Stress*, *36*(1), 60–85. 10.1080/02678373.2021.1936287

[CIT0029] Sonnentag, S., & Fritz, C. (2007). The recovery experience questionnaire: Development and validation of a measure for assessing recuperation and unwinding from work. *Journal of Occupational Health Psychology*, *12*(3), 204–221. 10.1037/1076-8998.12.3.204 17638488

[CIT0030] Sonnentag, S., Venz, L., & Casper, A. (2017). Advances in recovery research: What have we learned? What should be done next? *Journal of Occupational Health Psychology*, *22*(3), 365–380. 10.1037/ocp0000079 28358572

[CIT0031] Tennant, R., Hiller, L., Fishwick, R., Platt, S., Joseph, S., Weich, S., Parkinson, J., Secker, J., & Stewart-Brown, S. (2007). The Warwick-Dinburgh mental well-being scale (WEMWBS): Development and UK validation. *Health and Quality of Life Outcomes*, *5*(1), 1–13. 10.1186/1477-7525-5-63 18042300 PMC2222612

[CIT0032] Tims, M., Bakker, A. B., & Derks, D. (2012). Development and validation of the job crafting scale. *Journal of Vocational Behavior*, *80*(1), 173–186. 10.1016/j.jvb.2011.05.009

[CIT0033] Tims, M., Twemlow, M., & Fong, C. Y. M. (2022). A state-of-the-art overview of job-crafting research: Current trends and future research directions. *Career Development International*, *27*(1), 54–78. 10.1108/CDI-08-2021-0216

[CIT0034] Tušl, M., Bauer, G. F., Kujanpää, M., Toyama, H., Shimazu, A., & de Bloom, J. (2024). Needs-based job crafting: Validation of a new scale based on psychological needs. *Journal of Occupational Health Psychology*, *29*(2), 57–71. 10.1037/ocp0000372 38647461

[CIT0035] Tušl, M., De Bloom, J., & Bauer, G. F. (2022). Sense of coherence, off-job crafting, and mental well-being: A path of positive health development. *Health Promotion International*, *37*(6), daac159. 10.1093/heapro/daac159 36440899 PMC9703811

[CIT0036] Virtanen, A., De Bloom, J., & Kinnunen, U. (2020). Relationships between recovery experiences and well-being among younger and older teachers. *International Archives of Occupational and Environmental Health*, *93*(2), 213–227. 10.1007/s00420-019-01475-8 31552505 PMC7007884

[CIT0037] Weigelt, O., Gierer, P., Prem, R., Fellmann, M., Lambusch, F., Siestrup, K., Marcus, B., Franke, T., Tsantidis, S., Golla, M., Wyss, C., & Blume, J. (2022). Time to recharge batteries–development and validation of a pictorial scale of human energy. *European Journal of Work and Organizational Psychology*, *31*(5), 781–798. 10.1080/1359432X.2022.2050218

[CIT0038] Wickham, H., Averick, M., Bryan, J., Chang, W., McGowan, L. D., François, R., Grolemund, G., Hayes, A., Henry, L., Hester, J., Kuhn, M., Pedersen, T., Miller, E., Bache, S., Müller, K., Ooms, J., Robinson, D., Seidel, D., Spinu, V., … Yutani, H. (2019). Welcome to the tidyverse. *Journal of Open Source Software*, *4*(43), 1686. 10.21105/joss.01686

[CIT0039] Wrzesniewski, A., & Dutton, J. E. (2001). Crafting a job: Revisioning employees as active crafters of their work. *The Academy of Management Review*, *26*(2), 179–201. 10.5465/amr.2001.4378011

[CIT0040] Zhang, F., & Parker, S. K. (2019). Reorienting job crafting research: A hierarchical structure of job crafting concepts and integrative review. *Journal of Organizational Behavior*, *40*(2), 126–146. 10.1002/job.2332

